# Serum and Salivary Level of Nitric Oxide (NOx) and CRP in Oral Lichen Planus (OLP) Patients

**DOI:** 10.30476/DENTJODS.2019.77842

**Published:** 2020-03

**Authors:** Atena Shiva, Shahin Arab, Seyyed Jaber Mousavi, Ali Zamanian, Avideh Maboudi

**Affiliations:** 1 Dept. of Oral and Maxillofacial Pathology, Faculty of Dentistry, Mazandaran University of Medical Sciences, Sari, Iran; 2 Dept. of Clinical Biochemistry, Faculty of Medicine, Mazandaran University of Medical Sciences, Sari, Iran; 3 Dept. of Community Medicine, Faculty of Medicine, Mazandaran University of Medical Sciences, Sari, Iran; 4 Dept. of Restorative, Faculty of Dentistry, Mazandaran University of Medical Sciences, Sari, Iran; 5 Dept. of Periodontics, Faculty of Dentistry, Diabetes Research Center, Mazandaran University of Medical Sciences, Sari, Iran

**Keywords:** Nitric oxide, Inflammation, C-reactive protein, Oral lichen planus

## Abstract

**Statement of the Problem:**

Oral lichen planus (OLP) is a chronic inflammatory oral mucosal disease with unclear etiology while a few cases of disease become malignant.

**Purpose:**

This study aimed to evaluate the level of nitric oxide (NOx) and C-reactive protein (CRP) as oxidative stress and inflammation status in sample of OLP patients.

**Materials and Method:**

In this case-control study, serum and salivary NOx and CRP levels were evaluated in twenty two OLP patients as the case group confirmed by clinical and histopathological diagnosis, and twenty two healthy control groups collected from Tooba Oral Pathology Laboratory in Sari in 2016. The data were analyzed by using independent-samples t-test, Mann-Whitney U-test and Chi-square by using SPSS version 21. The statistical significant level was considered at *p*< 0.05.

**Results:**

Salivary and serum NOx levels in case group showed statistically significantly higher than healthy control group (*p*= 0.035 and *p*= 0.001, respectively). CRP values were significantly higher both in serum (*p*= 0.001) and in saliva (*p*= 0.035). A significant correlation was found between CRP and NOx values in serum (r= 0.521, *p*= 0.0001) and saliva (r= 0.427, *p*= 0.045).

**Conclusion:**

Oxidative stress causes damage to organs in the human body. Correct understanding of oxidative stress and its association with free radicals and inflammatory markers related to oral disease are important for effective treatments. The results of the study advocate the effects of NOx and CRP levels in pathogenesis of OLP. Therefore, antioxidant drugs might probably be considered in the treatment of OLP.

## Introduction

Oral lichen planus (OLP) is a common mucocutaneous lesion with chronic inflammatory progression that is presumably caused by activation of immune response to skin and mucous changes [ 1 - 2 ]. The oral lesions appear as white furrows or linear, papules, plaques, erythema, erosions or ulcers in the mouth and mostly affect the buccal mucosa, tongue, and gingiva. Currently, six clinical types of OLP have been reported including reticular, erosive, atrophic, papular, plaque-like and bullous [ 1 - 3 ]. The global prevalence of adult lichen planus (LP) ranges from 0.22 to 5% [ 4 ]. Concurrent oral and skin lesions have been observed in 70–77% of subjects with LP [ 5 ]. The prevalence of OLP has been reported to be 0.1 to 2.2%, though it is rare in children (less than 2-3%) [ 4 ]. 

The potential of OLP to change to carcinogenic condition is yet to be determined [ 2 ]. While the rate of malignant change of OLP has been reported to be 0.4-5%, the inherent risk for transformation to oral squamous cell carcinoma, as the most prevalent oral cancer (94%) remains poorly recognized [ 2 , 6 - 7 ]. The World Health Organization (WHO) describes OLP as a precancerous disorder, particularly concerning the erythematous (atrophic) and erosive forms [ 8 - 9 ]. The prognosis of oral SCC improves with early diagnosis and treatment, consequently, routine surveillance of OLP patients is imperative [ 2 ].

Free radicals are concerned for their role in inflammatory and autoimmune diseases. A range of reactive oxygen species (ROS) are produced by different cells such as keratinocytes, fibroblasts, and inflammatory cells [ 10 ]. The high production of these species may be a sign of cellular damage and offended anti-oxidative immune system. The imbalance between the systemic production of reactive oxygen and antioxidant or else to repair the consequential damage is explained as the oxidative stress [ 10 - 11 ]. ROS can be a central intermediary of damage to the cell structures, including lipids, membrane, proteins and DNA [ 12 ]. In the explanation of histopathologic forms of LP, basal cell generation, infiltration of inflammatory cells (like T lymphocytes) and damage of keratinocytes have been deliberated [ 1 ]. Free radicals and other active oxygen compounds exaggerate the inflammatory responses with participation of T lymphocytes and destruct the lipid membrane of keratinocytes [ 13 - 14 ]. Increase lipid peroxidation products and reduced antioxidant protective system has been reported on patients with genital lichen planus [ 10 ]. The effect of stress oxidative in pathophysiologic changes of basal cells of epidermis in LP patients has also been studied [ 10 ]. 

Nitric oxide (NOx) is a short living product of nitrogen metabolism, produced by many cells in the organism with very significant physiological function [ 15 - 16 ]. Endothelia and neural cells produce NOx; macrophages and other inflammatory cells can induce its synthesis and release. The most important inductors of NOx synthesis are bacterial products [ 17 ]. It has been recognized that biological functions of NOx can be separated into two categories. First, it acts as an endothelial-derived relaxer of vascular smooth muscle, an inhibitor of platelet aggregation and adhesion, and a neuronal messenger. Secondly, the NOx synthesized in large amounts by activated macrophage is a cytotoxic molecule influencing the ability of cells to kill bacteria, viruses and protozoa as well as tumors cells. Moreover, it is well established that NOx secreted by macrophages has damaging effects against cellular proteins, DNA, and lipids leading to periodontitis [ 18 ]. The synthesis of the CRP increases dramatically in acute phase of infection, but mildly elevated levels are also associated with chronic inflammation [ 19 ]. Since the etiology of LP is still unknown, the treatment of the disease would consequently be a suggestive approach [ 20 - 21 ]. The current study intended to evaluate the stress oxidative status and inflammation with the NOx index in a sample of Iranian population with OLP to verify the etiopathogenesis of the disease and therefore, proposing some suggestion to find an applicable treatment.

## Materials and Method

### Patient selection

In this case-control study, twenty-two patients (twelve women and ten men) with OLP, confirmed by clinical and histopathological diagnosis of previous oral biopsy were recruited. The study subjects were collected from patients referred to Oral pathology clinic, Mazandaran University of Medical Sciences and patients in a center of oral and maxillofacial pathology in Sari. A total of twenty-two healthy individuals (nine men and thirteen women) without any oral mucosal disease and matched to the case subjects were selected as control group from the same centers. This project has been approved by the Ethic committee of Mazandaran University of Medical Sciences (Code: 1048). Data were collected from all the patients in a structured questionnaire. Body mass index (BMI) was calculated as weight (Kg/height (m2^2^).The patients had a diagnosis of biopsy-proven reticular or popular OLP with a minimum of two microscopic criteria including a subepithelial lymphocyte band-like infiltrate and focal signs of basal layer hydropic degeneration detected by an oral and maxillofacial pathologist. In case group, samples untreated for the disease with the beginning signs within 3 months were recruited. Patients with lichenoid lesions associated with drugs or restitutions and other lichenoid lesions as well as presence of any dermatological involvement of OLP were excluded. Moreover, patients with positive history of candidiasis confirmed by laboratory assays and main systemic diseases were excluded from the study. All subjects in case and control group were medically examined for routine hematology and biochemical parameters (FBS, ESR, and liver profile) [ 21 ]. Those subjects consuming any oral medication that could positively influence the study parameters such as non-steroidal anti-inflammatory drugs, immunosuppressive agents or any vitamins supplements in the previous three months, and subjects with history of trauma or surgery, history of smoking or alcohol consumption (at least one month before the study) were excluded [ 11 , 21 - 22 ]. 

### Oral examination

Oral examination included examination of lips and oral cavity. To certify the disease, the presence of reticular and/or papular lesions consisting of lace-like and/or pinhead- sized, white, slightly elevated keratotic patterns in any location of the oral cavity were examined. All oral clinical examinations were performed by the same examiner, a specialist in oral and maxillofacial pathology [ 21 ].

### Salivary and serum tests

Approximately 10 ml fasting blood sample from case subjects and healthy control were taken into tubes. The serum was prepared, aliquoted and stored at -80 ο C until the analysis were carried out. Whole saliva samples were taken from patients and controls after fasting for at least twelve hours (after an all-night fasting) [ 20 , 23 - 24 ]. Before saliva collection, mouths were cleaned out with distilled water and unstimulated whole saliva was collected for 5 minutes with the subject leaning forward and spitting saliva into a graded sampling tube. As soon as saliva were collected, saliva samples were centrifuged at 900 g for 10 minutes at +4ο C, the upper parts were drawn and stored in small aliquots at- 80ο C until analyzed. All salivary samples were tested twice.

### NOx assay

The determination of NOx was based on the Griess reaction in which a chromophore with a strong absorbance at 545 nm is formed by reaction of nitrite with a mixture of N-naphthylethylenediamine and sulfanila-mide [ 24 ]. For NOx determination, aliquots of the sample were mixed with Cadmium in glycine buffer at pH 9.7 to reduce nitrate to nitrite, which was then mixed with fresh reagent. The absorbance was measured by spectrophotometer (UV-1800 Shimadzu double-beam) [ 24 ]. 

### CRP assay

CRP was measured by Selectra Flexor/XL auto analyzer (ELITech, France). This assay was based on the principle of particle-enhanced immunological agglutination or immunoturbidimetric assay. Following agglutination, this was measured turbidimetrically. The results of serum and saliva were expressed in (mg/L) and (μg/L) respectively [ 23 ]. 

### Data analyses

The results were presented as the mean±SD. Significant differences of normal distributed variables between 2 groups were assessed by independent-samples- t-test, while variables with non-Gaussian distribution were compared using nonparametric Mann-Whitney U-test to compare medians and the Chi-square test for categorical variables. The relationship between serum and saliva variables was assessed using Spearman’s rho correlation coefficient. *p*< 0.05 were assigned statistically significant. The SPSS version 18 was used for data analyzing. 

## Results

The mean age of the OLP patients and the control individuals were 48.7±9.2 and 43.7±6.9 years respectively without statistically differences (Table 1). Results in BMI did not show any statistically significant differences between OLP and healthy groups (Table 1). Salivary and serum NOx levels were shown statistically significantly higher than healthy control group (*p*= 0.045, *p*= 0.001). CRP value was significantly higher in serum (*p*= 0.001) and in salivary fluid (*p*= 0.035) (Table 2). In addition, statistically significant results were found in ESR in patients with OLP compared with healthy control group. Linear regression analysis showed no correlation between salivary and serum levels of CRP. A significant correlation was found between serum CRP and NOx value(r=0.521, *p*= 0.0001) and saliva (r= 0.427, *p*= 0.045). There were no significant differences in total protein and WBC count in case and control group. The NOx and ESR parameters were significantly associated with the tertiles of CRP value (Table 3).

**Table1:** Baseline characteristic of oral lichen planus and control group

Parameters		OLP group	Control group	*p*
Age(years)		48.7±9.2	43.7±6.9	0.243^a^
Sex	Male(n)	10	9	0.56^b^
	Female(n)	12	13	
BMI(Kg/m^2^)		25.12±4.16	26.11±2.47	0.524 a

esults are expressed as mean±SD

a student’s t- test,

b Pearson’s Chi-square test

SD: Standard Deviation, OLP: Oral Lichen planus, BMI: Body mass index

**Table2:** Biochemical and hematological parameters of oral lichen planus and control group

Parameters	OLP group Mean±SD	Control group Mean±SD	*p*< 0.05
CRP in Serum (mg/L)	3.24±2.44	1.72±1.14	0.001*
CRP in Saliva (μg/L)	12.4 ±5.8	10.7±3.6	0.035*
NOx in Serum (μmol/L)	55.81±20.34	35.54±7.08	0.001*
NOx in Saliva (μmol/L)	84.00±37.66	66.75±23.66	0.045*
Total Protein in serum (g/dL)	7.57±0.68	7.76±0.65	0.364
Total Protein in saliva (g/dL)	1.22±0.38	1.30±0.35	0.58
ESR (mmHg)	16.8±8.3	9.4±2.36	0.001*
WBC (10^3^/mm^3^)	6.8±3.4	5.6±3.6	0.264

* The p< 0.05 as compared to control

**Table3:** Data for quintile of C- reactive protein (CRP) variability

Range SD (C- Reactive Protein)	‹1.5 mg/L Mean±SD	1.5-3mg/L Mean±SD	›3 mg/L Mean±SD	*p*<0.05
NOx (μmol/L)	45.72±17.21	47.28±16.62	56.43±20.45	0.001*
ESR(mmHg)	9.2±3.4	14.4±7.6	16.7±9.4	0.001*

The cases were categorized into three groups: ‹1.5, 1.5-3, and ›3 mg/L based on CRP variability. The significant of differences as mean or median were tested using analyses of variance and Kruskal- Wallis test respectively.

* The *p*< 0.05 as compared to control

The associations of risk factors were performed between OLP patients and control group by using logistic regression analysis (Table 4). Biochemical parameters of CRP (serum and saliva), NOx levels (serum and saliva), and ESR parameter showed significant associations between these variables and OLP. These associations in CRP, NOx both in serum and ESR showed strong regression in adjusted odds ratio (Table 4).

**Table4:** Logistic regression analysis

Variables	OR (adjusted) Exp(β)	*p*	95%Cl
CRP in Serum(mg/L)	1.006	0.032	1.000-1.009
CRP in Saliva(μg/L)	1.595	0.040	1.012-2.514
NOx in Serum(μmol/L)	1.010	0.032	1.008-1,014
NOx in Saliva(μmol/L)	1.870	0.048	1.007-3.745
ESR(mmHg)	1.003	0.003	1.000-1.005

The Odds ratio for continuous variables are presented as the standardized regression coefficients as the term Exp (β); OR, odds ratio; Cl, confidence interval.

## Discussion

The present study confirms previous studies regarding the NOx plays several roles in both intracellular and extra cellular signaling mechanisms with implication for health and disease. Hence, increased level of NOx as a free radical has been proposed as a serious destructive mediator to the structures of cells, including lipids, proteins, and DNA that can be evident as oral ulcers [ 12 ]. Study of Bogdan *et al*. [ 25 ] confirmed the role of NOx as regulator in development, differentiation, and function of T and B-lymphocytes and increase T4 lymphocytes proliferations [ 26 ]. It has been hypothesized that radical NOx interacts with ROS and causes inflammatory responses.

ROS can be a central intermediary of damage to the cell structures, including lipids, membrane, proteins, and DNA [ 12 ]. In the current study, results in serum NOx levels in OLP patients were more than healthy control subjects. The findings yielded by this study are similar to the study of Mehdipour *et al*. [ 11 ] and Sunitha *et al*. [ 18 ] that reported increased levels of salivary NOx compared to the healthy individuals. Moreover, in the study conducted by Aly *et al*. [ 14 ] in Egypt, the serum level of NOx was substantially increased similar to the findings of our study [ 14 ].

On the other hand, the findings of the present study differ from the results of Brennan *et al*. [ 27 ] that failed to validate a significant difference in NOx synthetize staining between the patients and healthy control group.

In our recent published study, salivary and serum level of Malondialdehyde (MDA) and total antioxidant activity (TAA) in twenty-two patients with OLP was investigated and revealed significant differences with healthy control and confirmed that increased oxidative stress and lipid peroxidation might be involved in the pathogenesis of OLP [ 21 ]. Inflammation produces disturbance in lipid metabolisms and chronic inflammation contribute in increasing the risk of OLP [ 21 , 28 ]. However, Aghahosseini *et al*. [ 29 ], reported that the process of oxidative stress play an important role in pathogenesis of OLP. The findings of our study advocate that increased oxidative stress and increased lipid peroxidation, parallel with high levels of CRP, might be involved with the pathogenesis of OLP. Therefore, the results of the current study support the potential use of salivary and serum CRP as a parameter for systemic levels of inflammation [ 30 ]. The correlation of CRP levels with chronic inflammation is reported, which reveals the premalignant nature of lesion [ 27 ]. The results of the study conducted by Atas *et al*. [ 31 ] showed high levels of CRP and ESR with high neutrophil count, which might support the systemic inflammatory process in LP patients. Santiago *et al*. [ 32 ] showed patients with LP presented higher lipid peroxidation levels and CRP; chronic inflammation may elucidate these findings. 

 The hematological parameters of patients significantly differ from the age- and sex-matched normal subjects. Our results also revealed the possibility of the NOx and ESR parameters were significantly associated with the tertile of CRP value. The high level of CRP exhibited a strong association for both ESR and CRP level. However, it has been explained that both high ESR and WBC count are involved in etiology of OLP and may be recommended for assessment of the severity of the disease and treatment options [ 33 ]. In the present study, reticular and popular textures were evaluated; therefore, further studies with larger sample size and other clinical features of OLP are suggested to confirm the etiopathogenesis of the disease.

## Conclusion

The present results indicate correlation between serum and saliva NOx, CRP and other inflammation parameters in OLP patients. Oxidative stress causes damage to organs in the human body. Correct understanding of oxidative stress and its association with free radicals and inflammatory markers related to oral disease are import-ant for effective treatments. The results of the study sup-orted the effects of NOx and CRP levels in pathogenesis of OLP. Regarding these results, antioxidant drugs could probably be considered in the treatment of OLP. 

## References

[1] Nevil BW, Damm DD, Allen CM, Chi AC (2016). Oral and maxillofacial pathology.

[2] Shiva A, Zamanian A, Arab Sh, Boloki M ( 2018). Immunohistochemical Study of p53 Expression in Patients with Erosive and Non-erosive Oral Lichen Planus. J Dent Shiraz Univ Med Sci.

[3] Mortazavi H, Safi Y, Baharvand M, Rahmani S ( 2016). Diagnostic Features of Common Oral Ulcerative Lesions: An Updated Decision Tree. Int J Dent.

[4] Gorouhi F, Davari P, Fazel N ( 2014). Cutaneous and mucosal lichen planus: a comprehensive review of clinical subtypes, risk factors, diagnosis, and prognosis. Sci World J.

[5] Parashar P ( 2011). Oral lichen planus. Otolaryngol Clin N Am.

[6] Feller LL, Khammissa RR, Kramer BB, Lemmer JJ ( 2013). Oral squamous cell carcinoma in relation to field precancerisation: pathobiology. Cancer Cell Int.

[7] Kumar M, Nanavati R, Modi TG, Dobariya C ( 2016). Oral cancer: Etiology and risk factors: A review. J Cancer Res Ther.

[8] Schlosser BJ ( 2010). Lichen planus and lichenoid reaction of the oral mucosa. Dermatol Ther.

[9] Yardimci G, Kutlubay Z, Engin B, Tuzun Y ( 2014). Precancerous lesions of oral mucosa. World J Clin Cases.

[10] Sander CS, Cooper SM, Ali I, Dean D, Thiele JJ, Wojnarowska F ( 2005). Decreased antioxidant enzyme expression and increased oxidative damage in erosive lichen planus of the vulva. BJOG.

[11] Mehdipour M, Taghavi Zenouz A, Bahramian A, Gholizadeh N, Boorghani M ( 2014). Evaluation of serum nitric oxide level in patients with oral lichen planus. J Dent (Shiraz).

[12] Valko M, Leibfritz D, Moncol J, Cronin MT, Mazur M, Telser J ( 2007). Free radicals and antioxidants in normal physiological functions and human disease. Int J Biochem Cell Biol.

[13] Fu YC, Jin XP, Wei SM, Lin HF, Kacew S ( 2000). Ultraviolet radiation and reactive oxygen generation as inducers of keratinocyte apoptosis: protective role of tea polyphenols. J Toxicol Environ Health A.

[14] Aly DG, Shahin RS ( 2010). Oxidative stress in lichen planus. Acta Dermatovenerol Alp PanonicaAdriat.

[15] Sezer E, Ozugurlu F, Ozyurt H, Sahin S, Etikan I ( 2007). Lipid peroxidation and antioxidant status in lichen planus. Clin Exp Dermatol.

[16] Páli T, Bencsik P, Görbe A, Ferdinandy P, Csont T ( 2015). Measurement of NO in biological samples. Br J Pharmacol.

[17] Förstermann U, William C, Sessa A ( 2012). Nitric oxide synthases: regulation and function. Eur Heart J.

[18] Sunitha M, Shanmugam S ( 2006). Evaluation of salivary nitric oxide levels inoral mucosal diseases: a controlled clinical trial. Ind J Dent Res.

[19] Michael C, Dillon A, Daniel C, Opris A, Kopanczyk R, Lickliter J, Hayley N ( 2010). Detection of Homocysteine and C-Reactive Protein in the Saliva of Healthy Adults: Comparison with Blood Levels. Biomarker Insights.

[20] Michael C, Dillon A, Daniel C, Opris A, Kopanczyk R, Lickliter J ( 2010). Detection of Homocysteine and C-Reactive Protein in the Saliva of Healthy Adults: Comparison with Blood Levels. Biomarker Insights.

[21] Shiva A, Arab SH ( 2016). Evaluation of Uric Acid, Total Antioxidant and Lipid Peroxidation Parameters in Serum and Saliva of Patients with Oral Lichen Planus. Global Journal of Health Science.

[22] Al-Hashimi I, Schifter M, Lockhart PB, Wray D, Brennan M, Migliorati CA ( 2007). Oral lichen planus and oral lichenoid lesions: diagnostic and therapeutic considerations. Oral Surg Oral Med Oral Pathol Oral Radiol Endod.

[23] Shaheer KA, Tharayil KJ, Krishna WP ( 2017). A Comparative Study of High Sensitivity C-Reactive Protein and Metabolic Variables in Type 2 Diabetes Mellitus with and without Nephropathy. Journal of Clinical and Diagnostic Research.

[24] Hetrick EM, Schoenfisch MH ( 2009). Analytical Chemistry of Nitric Oxide. Annu Rev Anal Chem (Palo Alto Calif).

[25] Bogdan C ( 2011). Regulation of lymphocytes by nitric oxide. Methods Mol Biol.

[26] Niedbala W, Wei XQ, Campbell C, Thomson D, Komai-Koma M, Liew FY ( 2002). Nitric oxide preferentially inducestype 1T cell differentiation by selectively up-regulating IL-12 receptor beta 2 expression via cGMP. Proc Natl Acad Sci USA.

[27] Brennan PA, Umar T, Palacios-Callender M, Spedding AV, Mellor TK, Langdon JD ( 2000). A study to assess inducible with severity of chronic periodontitis. J Oral Pathol Med.

[28] Manisundar N, Julius A, Amudhan VA, Hemalatha, Manigandan T ( 2014). Nitric Oxide as an Inflammatory Biomarker in Oral and Systemic Diseases-A Systematic Review. Middle-East Journal of Scientific Research.

[29] Agha-Hosseini F, Mirzaii-Dizgah I, Mikaili S, Abdollahi M ( 2009). Increased salivary lipid peroxidation inhuman subjects with oral lichen planus. Int J Dent Hyg.

[30] Kumar ABS ( 2011). Altered C- reactive protein Levels in Serum of Oral Precancer Patients in Comparison With Healthy Controls. Int J Oral Maxillofac Pathol.

[31] Ataş H, Cemil BÇ, Kurmuş GI, Gönül M ( 2016). Assessment of systemic inflammation with neutrophil-lymphocyte ratio in lichen planus. Postepy Dermatol Alergol.

[32] Arias-Santiago S, Buendía-Eisman A, Aneiros-Fernández J, Girón-Prieto MS, Gutiérrez-Salmerón MT, Mellado VG ( 2011). Cardiovascular risk factors in patients with lichen planus. Am J Med.

[33] Abhishek J, Pratiti Gh ( 2014). Altered Hematological Profile of Oral Lichen Planus Patients. Research Journal of Pharmaceutical Biological and Chemical Sciences.

